# The influence of educational measures and low-level laser phototherapy on temporomandibular disorders: Study protocol clinical trial (SPIRIT Compliant)

**DOI:** 10.1097/MD.0000000000019005

**Published:** 2020-03-06

**Authors:** Caroline Maria Gomes Dantas, Carolina Lapaz Vivan, Solange Mongelli de Fantini, Patrícia Moreira de Freitas Costa e Silva, Claudio Mendes Pannuti, Andrea Lusvarghi Witzel, Gladys Cristina Dominguez

**Affiliations:** a Department of Orthodontics and Pediatric Dentistry; b Department of Restorative Dentistry, Co-Chairman of the Special Laboratory of Lasers in Dentistry; c Department of Stomatology, School of Dentistry - University of São Paulo, São Paulo, SP, Brazil.

**Keywords:** temporomandibular disorder, phototherapy, low-level laser therapy, multiple wavelengths, counseling, orofacial pain, pain pressure threshold, mandibular function

## Abstract

Photobiomodulation therapy (PBMT) with low-power laser is used for pain relief in several clinical conditions, including temporomandibular disorders (TMD). As musculoskeletal pain often produces changes in motor behavior, it is common for patients with TMD to present limited mandibular movements. To the date, there is no consensus about the optimal dosimetric parameters of PMBT for TMD. This randomized, controlled, double-blind clinical trial aims to evaluate pain relief and mandibular mobility in patients with TMD following treatments with 2 laser wavelengths, red (660 nm) and infrared (808 nm) individually and in combination as compared to a placebo treatment. One-hundred participants presenting myalgia and arthralgia, with disk displacement or not, will be selected based on the Research Diagnostic Criteria for Temporomandibular Disorders. All participants will be instructed about the etiology, prognosis, and self-care techniques for pain control on TMD, and followed up for 2 weeks. After this period, those who still present pain score over 4 in a visual analog scale (VAS) will be included in the study. Participants will be randomly assigned to 4 treatment groups: G1 = placebo (SHAM); G2 = PBMT with red laser (660 nm, 0.034 cm^2^, 88 J/cm^2^, 100 mW, 3 J/point); G3 = PBMT with infrared laser (808 nm, 0.034 cm^2^, 88 J/cm^2^, 100 mW, 3 J/point); and G4 = PBMT with red and infrared laser alternated between sessions. The treatment consists of 8 sessions, 2 times a week. The effect of the proposed therapies will be measured by: pain reduction in VAS; pressure pain threshold on TMJ, masseter and temporal muscles; and the amplitude of mandibular movements (opening, protrusion, and right and left lateral movements). The data will be collected at the following times: initial (T1), after the 1st treatment session (T2), at the end of treatment (T3), and 30 days after the last PBMT session (T4). For statistical analysis will be used 2-way repeated measures analysis of variance test, complemented by a post hoc Tukey test (*P* < .05).

## 1 Introduction

Temporomandibular disorder (TMD) is referred as the 2nd most frequent cause of orofacial pain among adults.^[1]^ It is estimated that 60% to 70% of the population has at least one sign of TMD, a condition that commonly affects the masticatory muscles, temporomandibular joint (TMJ), and associated structures.^[1]^ As TMD's frequent clinical manifestation, musculoskeletal pain is usually referred to the lateral area of the face, head, and cervical region.^[2,3]^ Pain often produces changes in motor behavior,^[4]^ so it is common that patients with TMD present limitations or restrictions on mandibular movements.^[5]^ Other recurrent symptoms are joint noises, tinnitus, and aural fullness.^[6]^ The literature also relates TMD pain with sleep disorders, fatigue, lack of concentration, mood alterations, and difficulties to perform daily activities.^[7]^

Historically, occlusal therapy with stabilization splints is referred to as the most commonly used therapeutic intervention for TMD management.^[8]^ However, there is a recurrent debate on the literature questioning the role of occlusion in the development of TMD.^[6]^ Thus, the vision on the complexity of this disorder was broadened and new therapeutic approaches have been incorporated into the field research.

Recent investigations show that the photobiomodulation therapy (PBMT) has become a useful therapeutic option in the management of musculoskeletal disorders.^[9–16]^ Its mechanism of action on TMD is still not completely known, but authors have reported the induction of analgesia, the stimulus to tissue healing and remodeling, the modulation of proinflammatory chemical mediators, the induction of muscle relaxation, and the dissolution of trigger points.^[17]^

Ferraresi et al,^[12]^ in a literature review, point out that PBMT using low power lasers may increase muscle metabolic activity and decrease fatigue during physiotherapy tests. Thus, PBMT can improve the motor activity of the stomatognathic system. Bjordal et al^[13]^ suggest that PBMT can significantly reduce pain and increase joint health in chronic disorders. Recently, Panhoca et al^[14]^ conducted an investigation with different types of light sources, irradiating the TMJ masseter and temporal muscles with red LED (630 ± 10 nm), infrared (850 ± 10 nm), and near-infrared (780 nm) lasers, bilaterally. The authors reported a reduction in pain and an improvement in mouth opening in all experimental groups, with no differences among them. However, a recent systematic review points to a great divergence regarding laser therapeutic parameters, which leads to a lack of consensus regarding the validity of the PBMT for TMD care.^[15]^

Xu et al^[11]^ conducted a systematic review with meta-analysis and affirmed that laser PBMT may effectively relieve pain and improve mandibular function in patients with TMD, although the available data is scarce and the quality of the evidence considered very low/moderate. Thus, it becomes clear that there is urgency for well-designed investigations, easy to be adapted to the clinical routine, in the quest for optimal parameters for PBMT in TMD.

The purpose of this randomized controlled trial is to analyze the influence of red or infrared wavelengths, and their combination, on pain and mandibular function of patients with TMD. This trial aims to provide a better understanding of the therapeutic responses that might be achieved by a clinical innovative yet simplified approach, contributing to the construction of effective, safe and low-cost comprehensive strategies in the conservative management of TMD.

## 2 Methods

### 2.1 Trial design

This will be a randomized, controlled, parallel, double-blind clinical trial.

### 2.2 Objectives

Primary objective:

- To evaluate the efficacy of different photobiomodulation protocols (red, infrared, and red associated with infrared laser) on pain of patients with TMD.

Secondary objectives:

- To assess the immediate and longitudinal impact of proposed treatments on pain and mandibular mobility.
- To relate pressure pain threshold (PPT) and pain under visual analog scale during a bilateral palpation examination of the TMJ, temporal and masseter muscles, at different times of the treatment.
- To verify, 30 days after concluding the PBMT, the duration of the therapeutic effects produced by the proposed protocols.

### 2.3 Setting of the study and recruitment

Volunteers seeking for treatment at the primary care clinic of the Interdisciplinary Orofacial Pain League (Department of Stomatology, School of Dentistry, University of São Paulo, Brazil) will be screened according to the inclusion and exclusion criteria. This investigation will be conducted under the Resolution 466/2012 (Brazil's National Health Council). Participants will sign an Informed Consent Form approved by the Research Ethics Committee of the School of Dentistry, University of São Paulo (protocol n. 2,201,761), assuring they will have their identity preserved and granting the management of any unintended effects of trial interventions without any costs.

This protocol for clinical trial is registered at Brazilian Clinical Trials Registry - REBEC (www.ensaiosclinicos.gov.br) as RBR-2bnc6y, first published April 12, 2018.

### 2.4 Eligibility criteria

Inclusion criteria: aged between 18 and 50 years; both genders; diagnosis of myalgia and arthralgia, with or without disk displacement; report of TMJ-related pain greater than score 4, measured with visual analog scale (VAS), after 2 weeks of guided self-care; and literate.

Exclusion criteria: patients with muco-supported dental prostheses, orthopedic, or orthodontic appliances; whose state of health contraindicates some of the therapies; presenting teeth in precarious conditions, for example, with indication of endodontic treatment; carriers of rheumatoid arthritis; who are using any analgesic, anti-inflammatory, antidepressant, and/or anxiolytic drugs; carriers of congenital problems with involvement of TMJ and/or cervical region; with recent mandibular fractures; pregnant women; and who are already being treated for TMD in another service.

### 2.5 TMD diagnosis and evaluation methods

A full anamnesis and physical examination (Research Diagnostic Criteria for Temporomandibular Disorders – RDC/TMD^[18]^) will be conducted to diagnose the type of joint and muscular impairment present. Pain levels will be recorded using a VAS and the PPT, with an algometer. The mandibular function will be assessed by the width of mouth opening, protrusion, and right and left lateral jaw movements. All clinical evaluations will be performed by a single experienced researcher (CMGD), blinded to group allocation.

- RDC/TMD: Participants will be examined seated in a dental chair, in a room with adequate lighting, according to RDC/TMD recommendations. This examination is considered the gold-standard for research in TMD and accesses essential outcomes such as the presence of muscle/joint pain, and measures of maximum mouth opening, protrusion, right and left lateral movement, which will be measured with a digital caliper.
- VAS: Each participant will be instructed to draw a mark on a 10-cm straight line with the endpoints defined as “no pain at all” and “worst pain imaginable”. The distance between “no pain at all” and the mark will define the subject's self-report of pain. Patients will be inquired for overall spontaneous pain, and the intensity of pain during palpation of the TMJ, anterior temporal, and masseter muscles bilaterally (according to the RDC/TMD instructions).
- PPT: To a more accurate record of the pain experience, a digital algometer (Pain Test FPX 25 Algometer; Wagner Instruments, Greenwich, USA) will be used to apply a controlled pressure at a 90° angle on the areas to be investigated (TMJ, anterior temporal, and masseter muscles bilaterally). This equipment has a circular probe of 1 cm^2^; the movement will be executed at a uniform and constant speed (0.5 kgf/cm^2^/s).^[19]^ Participants will be instructed to say “stop” at the point that the test becomes painful. The same anatomic area will be tested three consecutive times, and the average of the value displayed (kgf) will be recorded as the PPT.

### 2.6 Experimental groups

All participants will undergo guided self-care educational measures for pain control in TMD. They will be instructed about the etiology and prognosis of TMD, the importance of the suspension of parafunctional habits and physical therapy strategies to pain control in TMD (such as exercises and heat therapy),^[20–22]^ during one-a-one sessions by an experienced physician (CLV). All subjects will be warned to not take any analgesic or anti-inflammatory medication during the study and to inform the professional if they started any medical treatment. Those who succeed in following the instructions for 15 days and still return with pain over score 4 in VAS will be invited to join this study. The participants will be randomly allocated into 4 groups as described in Table 1.

**Table 1:**
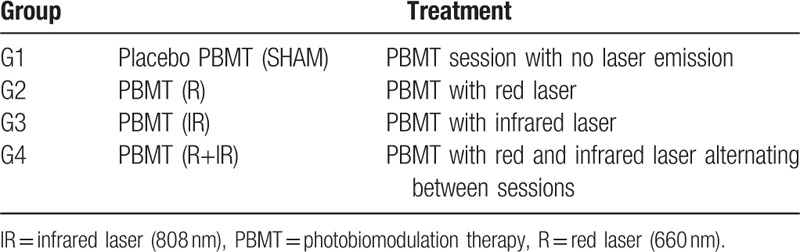
Experimental groups and correspondent treatment.

Once a participant is enrolled, the attendants (CMG, CLV) will make effort to assure patients retention for the entire study period with systematic methods for scheduling appointments and direct e-mail/phone contact as reminders for the consults.

### 2.7 Interventions

The 1st clinical session corresponds to a lecture on the etiology and prognosis of TMD, as well as recommendations for parafunctional habits avoidance and self-management of pain without medications.^[23–25]^ All participants will be followed-up for 2 weeks to check compliance with the educational measures and no-medication intake. Only the participants who still present pain in a VAS over score 4 after 2 weeks of self-management will be invited to join the study.

### 2.8 Protocols for PBMT

All participants will undergo PBMT according to the group to which they were allocated. The assigned group will be only revealed to the researcher responsible for the irradiation (CLV) by the time of the first PBMT session, after the 2-week educational measurements and initial evaluations (T1). The PBMT will be performed twice a week, totaling 8 sessions (4 weeks).

The G1 group (SHAM) will undergo a PBMT simulation. Negative control treatment will be held with a SHAM-equipment (DMC, São Carlos, Brazil) of the same design as the active equipment, simulating a PBMT session since it's able to emit an inert guide light (with no power), and reproduce the time marker's sounds identically to the active one.

The G2 group will be irradiated with an InGaAlP red laser (Therapy XT; DMC), 660 nm, 88 J/cm^2^, 100 mW, 30 s/point, spot size 0.034 cm^2^, 3 J/point, in continuous mode and with the tip positioned at 90° in contact with the skin.

The G3 group will be irradiated with an AsGaAl infrared laser (Therapy XT; DMC), 808 nm, 88 J/cm^2^, 100 mW, 30 s/point, spot size 0.034 cm^2^, 3 J/point, in continuous mode and with the tip positioned at 90° in contact with the skin.

The G4 group will be irradiated in the odd sessions with red laser (InGaAlP, 660 nm), following the G2 protocol; and in the even sessions, with infrared (AsGaAl, 808 nm) as described for the G3 protocol.

The equipment was previously calibrated and the output power is periodically checked using a power meter (Laser Check; MMOptics LTDA, São Paulo, Brazil). A summary of the irradiation parameters is shown in Table 2.

**Table 2:**
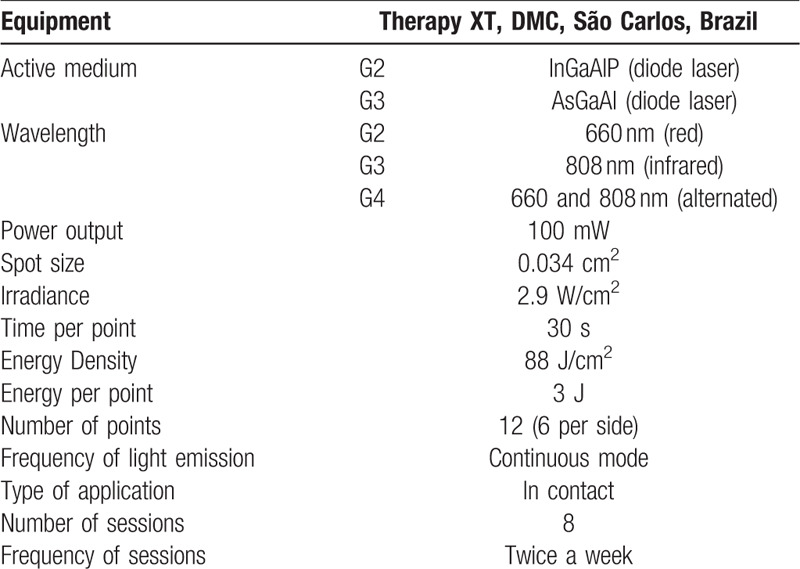
Photobiomodulation therapy protocols.

**Figure 1:**
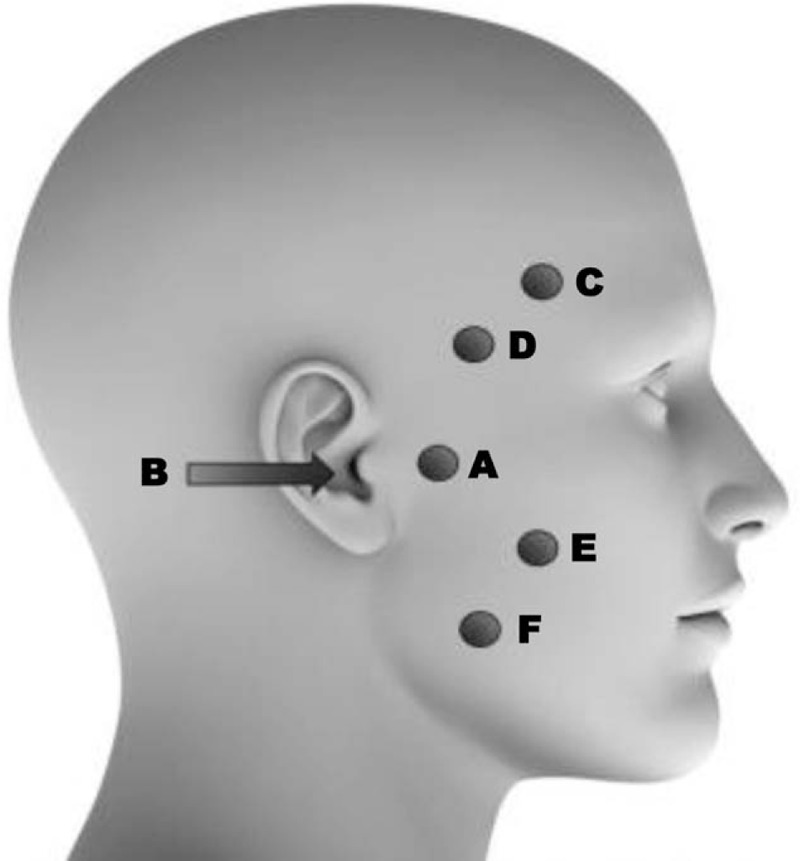
Irradiation points: anterior to the mandibular condyle (A) and intra-auricular toward the temporomandibular joint (B), 2 points on the superficial masseter muscle (C, D), and 2 points on the anterior temporal muscle bundle (E, F). Source: Adapted from mikomo/iStock.com.

To investigate the effect of the PBMT on painful experience around the TMJ, the laser light will be applied in 2 points: anterior to the mandibular condyle and intra-auricular toward the TMJ. The masticatory muscles will also be accessed, with a 2-point irradiation on the superficial masseter muscle and 2 points on the anterior temporal muscle bundle. The irradiation points will be equally distributed within the muscle area. The laser application points will be the same for all groups, bilaterally, and is exemplified in Figure 1. Safety measures for infection control, waste disposal, and protection from radiation by the operator and patient will be implemented in all therapy sessions.

### 2.9 Outcomes and data collection

Evaluations will be performed by a single trained examiner (CMGD) who is blinded to the allocation of the participants within the different treatment groups. Measurements will be performed at the following times: initial (T1), immediately after the 1st PBMT session (T2), at the end of the treatment (T3), and 30 days after the end of therapies (T4).

The primary outcome of the study will be pain reduction (baseline – 4 weeks) according to a visual analog scale of 10 cm. Pain reduction will be expressed in centimeter, as mean group values.

Secondary outcomes will be:

1. Pressure pain threshold in the TMJ, masseter, and anterior temporal muscles, bilaterally, with a digital algometer, registered in kgf (Pain Test Algometer FPX 25; Wagner Instruments).
2. Extension of mandibular range of movement, such as mouth opening (taking into consideration the overjet), protrusion (added the overbite), and right and left mandibular movement (noting possible midline deviation) in centimeter, with the aid of a digital caliper.

At each PBMT session, spontaneously reported adverse events and other unintended effects of trial interventions will be registered and managed according to the participant's need.

### 2.10 Sample size calculation

Sample size calculation was based on the primary outcome (mean pain reduction from baseline to 4 weeks examination), and based on ANOVA statistical test (4 groups). Considering an 80% power, alpha of 5%, and expecting a standard deviation of 2.5 cm, 23 individuals per group (totaling 92 participants) will be required to detect a minimum difference of 2.5 cm in VAS between the groups. In anticipation of a loss of 10% at follow-up, 100 study participants will be included (25 per group). This calculation was based on a study carried out in Brazil,^[16]^ in which the PBMT was effective in reducing the pain of individuals with TMD. The calculation aimed to detect the difference between the active laser groups and SHAM.

### 2.11 Assignment of interventions: allocation-sequence generation

Participants will be randomly assigned to one of the 4 groups with a 1:1 allocation as per a computer-generated randomization schedule using permuted blocks of 4 or 8 participants. The block sizes will not be disclosed to ensure concealment. The sequence generation was provided by one of the researchers (CMP) responsible for the study design but not directly involved with the subject's care.

### 2.12 Assignment of interventions: allocation-concealment mechanism and implementation

Once the participant is included in the study, the researcher responsible for the conduction of the PBMT will open a sequentially numbered, opaque, sealed envelope that reveals the participant's allocation group. The sealed opaque sequentially numbered envelope was provided by one of the researchers (SMF) responsible for the study design but not directly involved with the subject's care.

### 2.13 Blinding

All participants will undergo the same clinical conditions, and are therefore blinded to their allocation. Sham procedure will guarantee blinding of participants, as the equipment used to PBMT sessions for G1 has the same design as the active equipment, emits an inert guide light and the same noise as the laser equipment used to treat the other groups. The researcher (CMGD) responsible for measuring the study outcomes is also blinded to treatment allocation, characterizing the study as double-blinded. This professional will be responsible for feeding data into the computer in separate datasheets for further analysis without having access to information about the allocation at that moment.

### 2.14 Participant timeline

**Figure 2:**
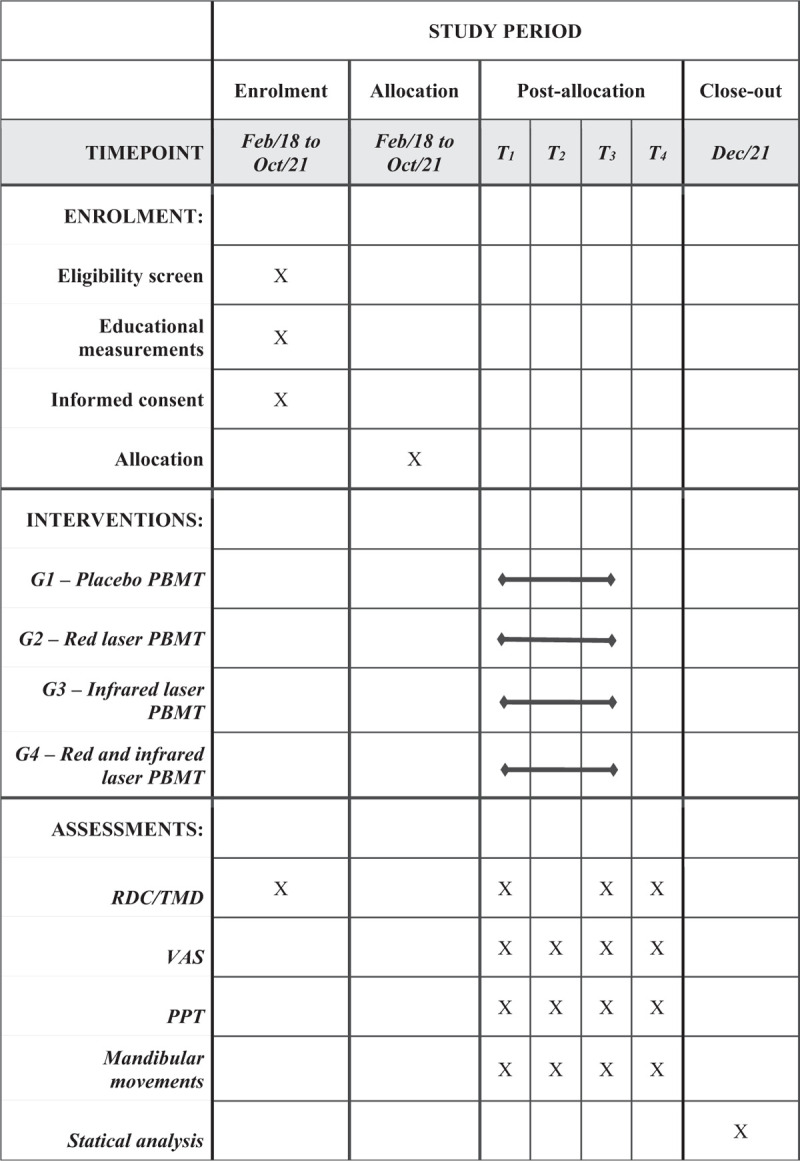
Schedule of enrolment, interventions, and assessments (SPIRIT 2013). PPT = pressure pain threshold, RDC/TMD = Research Diagnostic Criteria for Temporomandibular Disorders, TMD = temporomandibular disorder, VAS = visual analog scale.

Figure 2 shows participant timeline.

### 2.15 Statistical analysis

Statistical analysis will be conducted to compare the 4 groups of treatment, concerning the change from baseline of primary and secondary outcomes. Regarding the primary outcome, the groups will be compared using test of comparison of means. If there is adherence to a normal distribution and homogeneity of variances, the groups will be compared by analysis of variance (ANOVA; 2-way repeated measures ANOVA with post hoc Tukey Test). The analysis will be performed by intention to treat, with the imputation of data by carrying forward the last observed outcome in the case of missing data.^[26]^ Groups will be compared as regards other outcome using repeated measures ANOVA or a Chi-squared test. All tests will be conducted considering a 5% alpha.

## 3 Discussion

The search for conservative options to manage TMD's signs and symptoms has raised in the past years, but to the date, there is still no consensus about the optimal treatment protocol.^[20]^ Among the noninvasive modalities currently available, literature mostly highlights physiotherapy,^[21]^ the use of occlusal appliances,^[8]^ laser PBMT,^[9–16]^ and pharmacotherapy.^[22]^ These alternatives can be indicated isolated or in association with others. It is worth emphasizing that adjuvant treatments tend to reduce the demand for invasive and irreversible procedures, such as occlusal adjustments and surgical interventions.^[27]^

Medical therapies based on light sources are in ascendant focus as low-cost conservative healthcare methods. Light interaction and effects on biologic tissue depends on several variables, among them the wavelength of the laser. Although most of the studies in the field are based on infrared laser therapy, there is still no agreement on the appropriate wavelength for muscle and TMJ irradiation.^[28]^

As reported by Byrnes et al,^[29]^ infrared light between 770 and 850 nm is poorly absorbed on the surface of the skin, so it can be transmitted 8 to 10 mm through the tissue; as red light has a shorter wavelength, it's penetration tends to be smaller because of the greater interaction with chromophores at superficial levels.^[30,31]^ Still, considering the relatively superficial anatomical location of the TMJ, temporal and masseter muscles, it might be expected that both red and infrared laser radiation are able to reach the target tissues. Indeed, investigations varying the wavelength are justified by the superficial action of the red laser on superficial muscular bundles and deep action of the infrared laser on deeper muscular bundles, tendons, joints, and nervous tissue. Henceforward, although most articles report positive outcomes with infrared laser light for PBMT on TMD, few studies have shown good clinical results with the association of different wavelengths.^[32–34]^ Facing this gap, Maia et al^[35]^ call for caution on the interpretation of the available data and reinforce the need for investigations with a strong methodologic experimental design.

Another much-discussed topic is the mode and frequency of application for PBMT on TMD. The review by Herpich et al^[15]^ points out that the treatment protocols reported in the selected publications ranged from 1 to 20 laser sessions, with frequencies varying from 1 to 5 times a week. Furthermore, as for the irradiated area, some studies recommend laser application at predetermined points,^[19,36]^ others at acupuncture points,^[37]^ while some recommend the irradiation of trigger points.^[38,39]^ The evidence for the best laser application site and frequency for managing TMD's symptoms is insufficient, making it difficult to compare previously published protocols.

The effectiveness of PMBT for TMD has been considerably discussed^[11,15,35]^ mostly due the great divergence of the parameters used or insufficient data report constraining the clinician to replicate the PBMT protocol. Also, few clinical studies tested the performance of PBMT combining red and infrared laser irradiation for patients with TMD. This investigation will contribute to enlighten a evidence-based practice of PBMT for TMD, by reporting the effects of specific wavelengths on pain and mandibular function of individuals with myalgia and arthralgia.

A limitation of the study is that the care provider cannot be blinded to allocation, due to the nature of the intervention. Henceforward, this professional is instructed not to reveal the allocation status of the participant at the follow-up assessments, until separated datasheets for further analysis are complete.

Randomized clinical trials are the best source of scientific evidence to determine the efficacy of an intervention and serve as a reference for decision-making by health professionals. Clinical trials should be analytical, prospective, with the hypotheses to be tested described a priori, randomized, blinded and experimental to fulfill this referential function; therefore, the quality of these studies is of paramount importance.

### 3.1 Trial status

**Figure 3:**
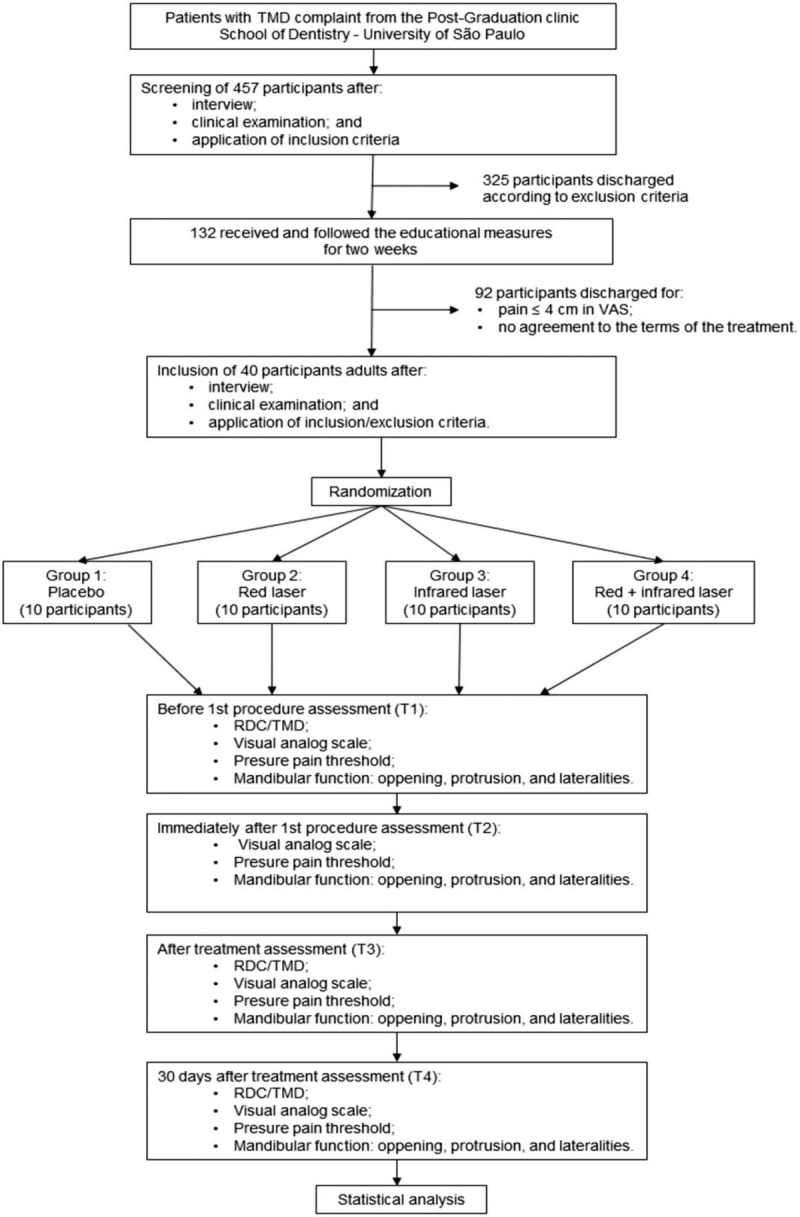
Patients flow chart. RDC/TMD = Research Diagnostic Criteria for Temporomandibular Disorders, TMD = temporomandibular disorder, VAS = visual analog scale.

Participants recruitment began at February 2018 and it is expected to end at October 2021. As shown on Figure 3, 1545 volunteers were screened, 457 participants fitted the study's inclusion criteria, 325 participants were discharged according to exclusion criteria, 132 received the educational measures, and only 40 were included in the study (34 women and 6 men, aged 19–43 years old, mean age 28.4 years). No side effects have been reported so far.

## Acknowledgment

The authors thank the Special Laboratory of Lasers in Dentistry (LELO-FOUSP) for their academic support and use of their equipment.

This study was financed in part by the Coordenação de Aperfeiçoamento de Pessoal de Nível Superior – Brasil (CAPES) – Finance Code 001.

## Author contributions

**Conceptualization:** Caroline Maria Gomes Dantas, Carolina Lapaz Vivan, Solange Mongelli de Fantini, Patrícia Moreira de Freitas Costa e Silva, Claudio Mendes Pannuti, Andrea Lusvarghi Witzel.

**Data curation:** Caroline Maria Gomes Dantas, Carolina Lapaz Vivan.

**Formal analysis:** Solange Mongelli de Fantini, Claudio Mendes Pannuti, Gladys Cristina Dominguez.

**Investigation:** Caroline Maria Gomes Dantas, Carolina Lapaz Vivan.

**Methodology:** Caroline Maria Gomes Dantas, Carolina Lapaz Vivan, Solange Mongelli de Fantini, Patrícia Moreira de Freitas Costa e Silva, Claudio Mendes Pannuti, Gladys Cristina Dominguez.

**Project administration:** Caroline Maria Gomes Dantas, Carolina Lapaz Vivan, Solange Mongelli de Fantini, Gladys Cristina Dominguez.

**Supervision:** Solange Mongelli de Fantini, Andrea Lusvarghi Witzel, Gladys Cristina Dominguez.

**Writing – original draft:** Caroline Maria Gomes Dantas, Carolina Lapaz Vivan.

**Writing – review & editing:** Caroline Maria Gomes Dantas, Carolina Lapaz Vivan, Solange Mongelli de Fantini, Patrícia Moreira de Freitas Costa e Silva, Claudio Mendes Pannuti, Gladys Cristina Dominguez.
